# Annexin A3 is a mammary marker and a potential neoplastic breast cell therapeutic target

**DOI:** 10.18632/oncotarget.4070

**Published:** 2015-06-04

**Authors:** Bashar Zeidan, Thomas R. Jackson, Samantha E.T. Larkin, Ramsey I. Cutress, Gary R. Coulton, Margaret Ashton-Key, Nick Murray, Graham Packham, Vassilis Gorgoulis, Spiros D. Garbis, Paul A. Townsend

**Affiliations:** ^1^ Cancer Sciences Unit, University of Southampton, Southampton, UK; ^2^ Faculty Institute for Cancer Sciences, Manchester Academic Health Sciences Centre, University of Manchester, Manchester, UK; ^3^ St George's Medical Biomics Centre, St. George's University of London, London, UK; ^4^ Molecular Carcinogenesis Group, Department of Histology and Embryology, School of Medicine, University of Athens, Athens, Greece; ^5^ Manchester Centre for Cellular Metabolism, University of Manchester, Manchester Academic Health Science Centre, Manchester, UK

**Keywords:** annexin A3, migration, neoplasm, biomarker, breast cancer

## Abstract

Breast cancers are the most common cancer-affecting women; critically the identification of novel biomarkers for improving early detection, stratification and differentiation from benign tumours is important for the reduction of morbidity and mortality.

To identify and functionally characterise potential biomarkers, we used mass spectrometry (MS) to analyse serum samples representing control, benign breast disease (BBD) and invasive breast cancer (IDC) patients. Complementary and multidimensional proteomic approaches were used to identify and validate novel serum markers.

Annexin A3 (ANX A3) was found to be differentially expressed amongst different breast pathologies. The diagnostic value of serum ANX A3 was subsequently validated by ELISA in an independent serum set representing the three groups. Here, ANX A3 was significantly upregulated in the benign disease group sera compared with other groups (*P* < 0.0005).

In addition, paired breast tissue immunostaining confirmed that ANX A3 was abundantly expressed in benign and to a lesser extent malignant neoplastic epithelium. Finally, we illustrated ANX A3 expression in cell culture lysates and conditioned media from neoplastic breast cell lines, and its role in neoplastic breast cell migration *in vitro*.

This study confirms the novel role of ANX A3 as a mammary biomarker, regulator and therapeutic target.

## INTRODUCTION

Breast cancer is the most common malignancy and the second leading cause of cancer related death in women [1–3]. Every year nearly 1.4 million women are diagnosed with the disease [4]. Early diagnosis and more effective treatment regimens have led to a paradoxical improvement of survival rates despite an increasing breast cancer incidence over the last few decades [5–7]. Nonetheless, a reported variation in screening mammography sensitivity between 74–85% suggests limitations to this technique [8]. Although screening programs lead to an estimated 15% reduction in breast cancer mortality, they were subsequently shown to be associated with 30% risk of over-diagnosis and treatment [9–11]. Prediction and stratification of breast disease at an early stage would further improve patients’ outcome and quality of life. In particular, early diagnostic markers allowing personalised management for women at the greatest risk are needed. Markers that reduce the incidence of “missed” or delayed breast cancer diagnosis would reduce the rate of excessive surgical and percutaneous benign biopsies thus improving diagnostic pathways, screening programs and patients’ well-being. Existing biochemical markers are also of limited value in assessing and stratifying breast cancer risk in the healthy population [12–15].

Molecular profiling studies are anticipated to improve the understanding of biological expression and characterization of cancer patients according to their individual risk of disease development, progression and therapeutic response [16]. This will also aid the development of novel multimodal therapeutic strategies improving disease outcome. Proteins are the main biological effectors in normal and cancer cells and hence can potentially serve as functional biomarkers and treatment targets. As such, proteomic profiling constitutes an ideal tool in the identification of key molecular evidence supporting current clinical practices.

In this study we sought to identify new diagnostic and stratification biomarkers of breast cancer by serum proteomic profiling. Our primary aims were to identify novel discriminatory biomarker(s) between age-matched groups representing controls, benign breast disease, and invasive ductal carcinoma of the breast, and to determine their potential clinical utility. Using a combination of mass spectrometry (MS) methods in an intra-laboratory setting with ELISA validation analysis, we identified a potential novel serum marker, Annexin A3. Paired breast tumour tissue immunostaining analysis confirmed the validity of this marker at the tissue level. Finally, we confirmed that ANX A3 was expressed and regulated migration in neoplastic breast cell lines. This study provides insight into a potential novel marker of breast tumourigenesis and its prospective role in clinical diagnostics and therapeutics.

## RESULTS

### Biomarker discovery and validation

MS profiling was conducted in two centers adopting MALDI-TOF MS-MS, SELDI-TOF MS, Infusion FT-Orbitrap MS^2^ and Ion-trap LC-MS^2^ analyses. The initial statistical analysis was performed as an independent single institute study and MS signals that demonstrated significant fold-change differences were cross-compared between the two institutes. A total of 1041 samples including 630 healthy controls, 219 invasive ductal carcinoma (IDC) and 192 benign breast disease (BBD) were analysed between the two sites.

**Figure 1:**
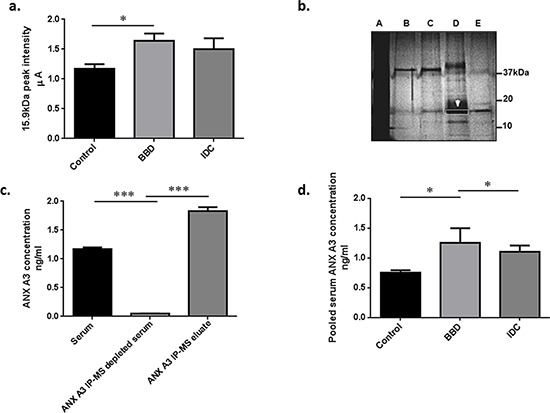
Bottom up proteomic analysis identified ANX A3 as a potential breast cancer marker **a.** Semi-quantitative MS expression profiling analysis was performed on serum samples in two centres (UoS and SGUL). Bars represent mean peak intensity ± standard error (SE) for the 15.9 kDa putative peak marker detected by MS analysis. Significant biomarker up-regulation in the benign disease group compared to controls and non-significant higher levels as compared to cancer sera fractions were illustrated. (Mann-Whitney *U* test *P < 0.05) **b.** Unfractionated serum (A), a series of different consecutively pooled and vacuum dried FFE fractions (B–E) were run on 1D-GE (4% tricine/10% SDS/16% polyacrylamide) gel at 100 V for 4 h at room temperature to purify the candidate BBD biomarker at 15.9 kDa. This approach was used to increase the biomarker concentration and reduce sample complexity. The dominant 15.9 kDa protein band (white arrow/box) was identified by infusion FT-Orbitrap MS^2^ and Ion-trap LC MS^2^ as ANX A3. **c.** IP-MS verification of the 15.9 kDa marker identified as ANX A3. Antibody capture of ANX A3 was performed using monoclonal antibody (sc-134260, Santa Cruz). The antibody was linked to μMACS protein A/G columns (Miltenyi Biotec) and incubated with pooled serum fractions containing ANX A3. The bars illustrate MS profiles of ANX A3 expression in serum fraction, along depleted specimen and the enriched ANX A3 IP eluate respectively. The depletion of ANX A3 in the IP MS samples and subsequent enrichment in the eluate spectra confirmed the identity of the 15.9 kDa peak as ANX A3. **d.** ANX A3 levels in pooled serum fractions measured by quantitative ELISA. ANX A3 over expression in control (*n* = 5) compared to both benign disease (*n* = 4) and invasive breast cancer (*n* = 5) groups was illustrated in this independent validation cohort. Bars represent the mean ANX A3 levels ± standard error (SE). *P < 0.05.

MS analyses detected an ion peak at *m/z* 15.9 kDa which was over-expressed in the benign disease compared to the healthy control group (*P* = 0.019) (Figure 1a). The intensity of this ion was observed to be stronger in the benign disease compared to the IDC group, however, significant differential expression was not detected between either the IDC versus BBD (*P* = 0.43), or the IDC versus control groups (*P* = 0.90).

### Biomarker purification, identification and verification

ANX A3 was identified as the 15.9 kDa marker following two dimensional purification using free flow electrophoresis isoelectric focusing (FFE-IEF) and one dimensional gel electrophoresis (SDS PAGE), tryptic digestion and sequencing of the equivalent 15.9 kDa gel band (Figure 1b and Supplementary Files S2, S3).

To further verify the ANX A3 identity, immunoprecipitation (IP) was performed to deplete ANX A3 from the test serum sample, and was then followed by MS analysis of the resulting extract. The *m/z* peak at 15.9 kDa was absent from the ANX A3 depleted sample and highly enriched in the ANX A3 IP eluate confirming the identity of this biomarker as ANX A3 (Figure 1c).

### Immuno-validation of ANX A3 expression in serum

**Figure 2:**
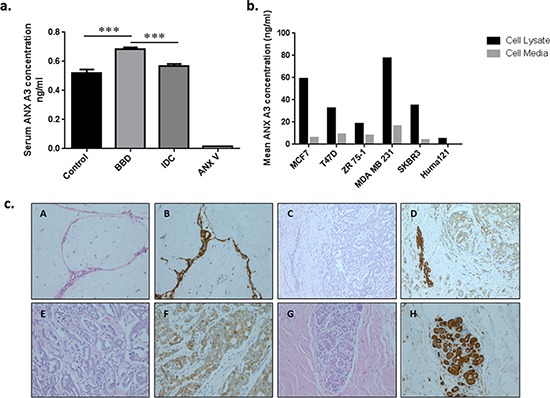
Immuno-validation and tissue correlation of ANX A3 **a.** ANX A3 levels in pooled serum fractions measured by quantitative ELISA. ANX A3 over expression in benign disease (*n* = 21) compared to both control (*n* = 14) and invasive breast cancer (*n* = 16) groups was illustrated in this independent validation cohort. Bars represent the mean ANX A3 levels ± standard error (SE). The ANX A3 ELISA kit detection specificity was assessed using ANX V recombinant protein as a negative control. Here, serum ANX A3 was significantly over expressed in the BBD compared to the control and IDC groups. ***P < 0.0005. **b.** Annexin A3 levels in the cell lysates (black bars) and culture medium (white bars) from six neoplastic human breast cell lines were measured by ELISA. ANX A3 expression was confirmed by ELISA in malignant cell lines (MCF7, ZR 75–1. T47D, SKBR3, MDA MB231) and conditioned media. A lower ANX A3 expression was detected in the non-malignant Huma121 cells and conditioned media. **c.** Representative photomicrographs of Annexin A3 immunohistochemistry in paired breast tissue corresponding to serum samples used in the MS analysis. A and B represent fibroadenoma (original magnification x200) with strong epithelial ANX A3 staining (B) C and D represent invasive carcinoma (x200) where the tumour is surrounding a benign duct. The tumour shows weak tumour ANX A3 expression compared to the benign area. E and F High power (x400) images of invasive ductal carcinoma showing weak annexin expression. G and H normal breast lobule demonstrating annexin expression. Overall, the BBD tissue ANX A3 staining was significantly higher than the other groups (*P* < 0.05).

To further assess the role and performance of ANX A3 as a clinical marker, ELISA validation of representative pooled serum samples from each group (*n* = 14) followed by additional randomly selected serum samples (*n* = 51) were conducted. The pooled serum ELISA validation confirmed significant ANX A3 overexpression in BBD compared to both IDC and controls (*P* < 0.05) (Figure 1d). Moreover, the individual serum sample ELISA validation has also confirmed ANX A3 levels to be higher in benign disease compared to the healthy control samples (*P* < 0.0005), consistent with our MS findings. Here, the ANX A3 levels were also significantly raised in benign disease compared to invasive breast cancer patients (*P* < 0.0005) (Figure 2a.). The levels of serum ANX A3 in benign disease (0.915 ng/ml, 95% CI 0.56 – 0.87) was significantly higher than both control (0.54 ng/ml, 95% CI 0.23 – 0.84), and invasive breast cancer (0.57 ng/ml, 95% CI 0.44 – 0.69) groups.

### ANX A3 expression in breast cancer and benign breast human cell lines

We tested the hypothesis that breast tumour cells secreting ANX A3 could explain the raised ANX A3 levels in benign and malignant breast neoplasms. Here, we used ELISA to quantify ANX A3 expression and secretion in neoplastic breast cell lines. The ANX A3 quantification confirmed that MCF7, T47D, MDA MB231, SKBR3, ZR 75–1 and HUMA121 human breast cells expressed and secreted ANX A3 (Figure 2b).

### Abundant ANX A3 expression in benign breast disease tissue samples

We next investigated ANX A3 expression at the tissue level by immunohistochemistry (IHC) using paired breast tissue samples analogous to sera used in the MS analysis. We analysed 27 randomly selected breast tissue samples corresponding to serum samples used in the earlier MS analysis (ten cancer, eleven benign disease and six normal breast tissue). Two histopathologists performed a blinded analysis independently using a semi quantitative scoring system (ANX A3 index) based on the percentage and intensity of IHC staining.

The IHC analysis showed that ANX A3 expression was predominantly cytoplasmic and was substantially over-expressed in benign breast disease and to a lesser extent in invasive breast cancer tissue compared with healthy controls (χ^2^ = 6.12, *P* = 0.045) (Figure 2c). Here, 66% of healthy breast tissue samples showed weak or absent ANX A3 staining. In comparison, 91% of benign breast disease tissue samples stained highly for ANX A3. Among malignant disease cases, 30% of cancer tissues had high ANX A3 expression profiles with the remaining cases having moderate or no ANX A3 expression.

### ANX A3 promotes cell migration in MCF-7 cells

**Figure 3:**
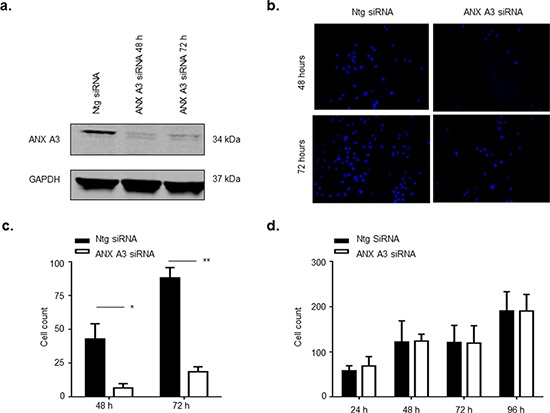
ANX A3 silencing inhibits migration of MCF7 cells MCF7 cells were transfected with 10 nM ANX A3-siRNA using Oligofectamine™ (Invitrogen) as per manufacturer's instructions. **a.** At indicated time points cell lysates were assessed by immunoblotting for ANX A3 and GAPDH as a loading control. The signal was analysed using a LICOR Odyssey imaging system. ANX3 was successfully silenced for the duration of the experiment. **b.** ANX A3 was silenced in MCF7 cells and cells were seeded onto the upper layer of the transwell cell permeable membrane transwell. At indicated time points cells were fixed, cells on the upper layer of the transwell membrane were removed whereas cells that had migrated to the underside of the transwell membrane were stained with DAPI stain and counted. ANX A3 visibly reduced the number of cells that migrated to the underside of the transwell membrane. **c.** Quantification of migration showed that migration was significantly reduced in ANX A3-silenced cells at both 48 h (*p < 0.01, *n* = 3) and 72 h (*P < 0.001, *n* = 3). **d.** 5000 cells were seeded into wells of 24 well plates, fixed at indicated time points and stained with DAPI central fields of view were images and cells counted. No difference in proliferation was observed between ANX A3silenced cells and control cells treated with ntg-siRNA (*n* = 3).

We investigated the effect of silencing ANX A3 on cell proliferation and migration in the MCF7 breast cancer cell line. Silencing of ANX A3 was achieved using ANX A3 siRNA (Figure 3a). ANX A3 silencing significantly inhibited the ability of MCF7 cells to migrate across the trans-well membrane (Figure 3b & 3c). However, ANX A3 silencing did not affect cells proliferation. This confirmed that the observed decrease in migrating cells was not due to an anti-proliferative effect (Figure 3d).

## DISCUSSION

In this study we mined for potential novel breast cancer serum biomarkers. Multi-platform MS analyses and validation of matched serum samples representing healthy volunteers, benign breast disease and invasive breast cancer groups was adopted to minimize false positive discovery risk. Following an extensive verification phase, a potential new breast cancer serum biomarker was detected and subsequently identified as human Annexin A3 (ANX A3), a phospholipid and calcium binding protein. This result was confirmed by ELISA validation analysis involving serum samples from an independent cohort representing the same three subject groups.

Current serum and hormonal markers for breast cancer are non-ideal and there is a need for reliable serum biomarkers for the prediction, diagnosis and stratification of breast tumours. Proteomics can significantly contribute in this regard. This study constitutes the first reported identification of higher levels of serum ANX A3 in BBD compared to both controls and invasive breast cancer patients. Screening mammography is reported to result in increased benign breast biopsies and some of these fail to confirm cancer [17–19]. Moreover, higher total biopsy rates are not associated with improved cancer detection [20]. In this context, serum ANX A3 levels could potentially complement and enhance mammographic screening performance and reduce the incidence of benign biopsies.

Annexin A3 is a member of a structurally homologous, but nonetheless functionally diverse annexin family [21]. ANX A3 is proposed to play anti-apoptotic, angiogenic and growth regulatory roles [22–24]. In addition, ANX A3 was shown to be differentially expressed in different malignancies in a tissue specific manner [25, 26]. Albeit an early finding, this work provides new evidence that ANX A3 expression may shed new light on the underlying biology of human breast neoplasms.

In this study, the majority of the benign breast disease samples represented non-proliferative breast conditions. Although the study design and cohort number are both limiting factors in evaluating the role of ANX A3 in reducing benign breast biopsies rate, as such, this could be a venue for further exploration. An interesting phenomenon demonstrated in this work was the over expression of ANX A3 in benign breast conditions as well as breast cancer samples compared to healthy women. Here, the benign disease group ANX A3 expression was shown to be higher than the cancer group both on the serum and tissue levels. The cancer serum and tissue samples on the other hand had higher ANX A3 expression compared to controls. Although the molecular mechanism explaining such trend remains unclear, a similar benign disease tissue ANX A3 over expression trend was found in benign prostate hypertrophy (BPH) relative to prostatic in situ neoplasms (PIN) and invasive prostatic carcinoma tissue samples [27, 28]. Secreted ANX A3 has also been shown to correlate with ovarian cancer chemotherapy resistance [29]. The specific molecular etiology of ANX A3 regulation in hormone-associated cancers thus warrants further evaluation. Equally, the expression behavior of ANX A3 in ductal carcinoma in situ (DCIS) and different IDC molecular sub groups (basal, ERBB-2 and luminal) necessitates larger sub-entity focused studies.

Here, we also demonstrated a similar ANX A3 over expression in benign and malignant breast tissue correlating to serum samples from this cohort. Although the over expression in malignant tissue immunostaining was more pronounced compared to the serum, serum is a multi-parametric reservoir of multiple organ systems that undoubtedly introduce several confounding factors negating its absolutely linear correlation with the tissue findings. The illustration of a consistent expression trend among the groups on the serum and tissue levels is intriguing. Based on our previous results, we hypothesized that elevated serum levels of ANX A3 protein were due to its increased secretion from neoplastic breast cells into the systemic circulation. To support this hypothesis, six neoplastic mammary cell lines were confirmed to consistently secrete ANX A3 as detected in their lysates and their supernatant culture media. Although the profiles of cell expression/secretion *in vitro* are rather difficult to compare to the *in vivo* cellular behavior, such consistency in expression/secretion trend across several neoplastic cell lines is more likely to reflect a candid biological path. The potential functional role of ANX A3 in mammary malignant transformation and/or progression is interesting as ANX A3 was defined previously as an independent prognostic factor related to neoplastic progression [30]. In addition, ANX A3, along with ANX A2 were found to be marker components of exosomes secreted by neuroblastoma cell lines [31]. This observation further strengthens the notion that ANX 3 is secreted and enriched by exosome mediated processes.

Finally, the potential functional role of ANX A3 in neoplastic breast activity was illustrated by halted migration despite maintained proliferation in the ANX A3 silenced MCF7 cell line. Although this evidence is of limited translational value at this stage, together with recent evidence of a neoplastic relevance of ANX A3 in breast cancer [32], it denotes another significant functional indication of a tumourigenic role of ANX A3 in breast cancer. As such, ANX A3 could be a potential therapeutic target decelerating breast cancer metastasis. Exploring the ANX A3 activity in various breast cancer cell lines representing the disease sub groups is deemed essential.

In conclusion, the present study provides more evidence of a potential role for ANX A3 as a serum and tissue breast cancer marker. ANX A3 is expressed and secreted by neoplastic mammary cells, and its inhibition halts migration in breast cancer cells. Large scale prospective studies together with long term follow-up and detailed molecular analysis are required to elucidate the role and mechanism(s) by which ANX A3 might impact breast pathology, diagnostics, and tumourigenesis.

## MATERIALS AND METHODS

### Patients

Serum samples were collected as part of the proteomic analysis in a breast screening study involving breast disease and healthy volunteers from the Wessex region (UK) prior to any intervention. Sample collection was approved by the Southampton General Hospital NHS Trust Ethics Committee (Ethical Approval 05/Q1702/13, R&D reference RHMCAN0392) and informed consent was obtained from all participants in the study. Samples from 630 controls, 192 benign breast disease (BBD) and 219 invasive breast cancer (IDC) patients were used in this study.

### Serum proteomic profiling and biomarker identification

Both the SELDI and MALDI-TOF MS profiling platforms were used for the MS profiling analysis and biomarker validation. Multidimensional proteomic biomarker purification, identification and verification using free flow and gel electrophoresis, FT Orbitrap and LC MS/MS sequencing were then implemented for *bona fide* identification of potential biomarkers as described in Supplementary Materials and Methods (File S1).

### ANX A3 ELISA and immunohistochemistry validation

ANX A3 measurements in serum fractions and cell lysate/media were performed using commercially available sandwich ELISA kits according to the manufacturers’ protocol (USCN Life Science, E94786Hu and CUSABIO Biotech Co. CSB-E12157 h). In addition, ANX A3 expression in formalin fixed paraffin embedded breast tissue blocks was performed by two pathologists applying an ANX A3 score as described in the Supplementary Materials and Methods.

### Evaluation of the molecular role of ANX A3 in breast cells

Six neoplastic human breast cell lines (HUMA121, MCF7, T47D, MDA MB231, SKBR3 and ZR 75–1) representing different molecular subgroups of breast neoplasms were cultured to analyse their ANX A3 expression and secretion. Moreover, ANX A3 expression was manipulated in MCF7 cells to assess its potential effect on mammary tumourigenesis.

## APPENDIX A SUPPLEMENTARY DATA


