# Supplementary material for: Annexin A3 is a mammary marker and a potential neoplastic breast cell therapeutic target
Source: Oncotarget. 2015 Jun 4;6(25):21421–7. doi: 10.18632/oncotarget.4070 (PMC4673275; doi:10.18632/oncotarget.4070)
Supplement: Supplementary file 1 [file oncotarget-06-21421-s001.pdf]

APPENDIX A SUPPLEMENTARY DATA

FILE S1: SUPPLEMENTARY MATERIALS AND METHODS

Serum sample collection and preparation

Serum samples were collected as part of the proteomic analysis in breast screening study involving breast disease and healthy volunteers from the Wessex region (UK) prior to any intervention (Table 1). Sample collection was approved by the Southampton General Hospital NHS Trust Ethics Committee (Ethical Approval 05/Q1702/13, R&D reference no. RHMCAN0392) and informed consent was obtained from all participants in the study.

Serum samples were fractionated using solid anion exchange (SAX) chromatography and pH gradient elution (pH between 9 and 2) as per manufacturer's instructions (BioRad, Hercules, CA, USA; Cat No. K10–00007). Protein array preparation for MS profiling was carried out using a BioMek3000 (Beckman Coulter, High Wycombe, UK) liquid handling robot where samples were applied to protein arrays using two technical replicates in a randomised fashion. Weak cation exchange (WCX) protein arrays were treated with 150 µl aqueous sodium acetate (50 mM, pH 4.0) twice for a total of 10 minutes at room temperature. An aliquot of 10 µl -mixed with 90 µl sodium acetate (50 mM pH 4.0) buffer to generate 1:10 dilutions-from each fraction was added to each spot. Samples were incubated for 60 minutes in a humid chamber to prevent drying at room temperature (25°C). Following incubation, arrays were washed with 150 µl ammonium acetate (50 mM, pH 4.0) buffer twice, rinsed with de-ionised water and allowed to air dry. Two applications of 1 µl of 50% sinapinic acid (SPA) in 50% acetonitrile (ACN) 0.5% trifluoroacetic acid (TFA) were added to each protein sample on the chip and allowed to air dry for 15 minutes at room temperature (25°C).

SELDI and MALDI-TOF MS-based proteomic profiling analysis

Both the SELDI and MALDI-TOF MS profiling platforms were used for the MS profiling analysis. For the SELDI-TOF MS analysis; protein arrays were analyzed using the Enterprise 4011 SELDI platform (BioRad, Hercules, CA). Data was analyzed using ProteinChip Data Manager v. 3.0.7 Software.

For the MALDI-TOF MS-MS analysis; protein arrays were analysed by MALDI-TOF MS Ultraflex III (Bruker Daltonics, Bremen, Germany). Spectra were acquired on the Ultraflex mass spectrometer in the linear mode at 80% laser power over a range of 2.5–100 kDa for a total of 200 laser shots using FlexControl 2.4 software (Bruker Daltonics, Bremen, Germany). Data was then analysed using Lucid system software (BioRad, Hercules, CA, USA).

The following settings were followed in both MS readers: Target *m/z* 5 kDa, matrix attenuation at 2.5 kDa and mass range between 0–100 kDa. External calibration was performed using protein standards comprised of recombinant hirudin (6.96 kDa), equine cytochrome *c* (12.23 kDa), equine myoglobin (16.95 kDa), and carbonic anhydrase (29.00 kDa). Mass accuracy (m/ßm) was calculated at $\leq$ 0.02% throughout the entire experimental mass range. Noise definitions were adjusted to eliminate chemical noise in the low mass range, the area below the detector blinding setting (*m/z* 2, 500) was excluded. Only peaks with a signal-to-noise (S/N) ratio of $\geq$ 5 and a valley depth $\geq$ 3 were considered for clustering. Qualified peaks which were present in $\geq$ 10% of the spectra were used to generate peak clusters. The mass window for each cluster was set at 0.3% of the peak mass for spectra optimized for low mass (0- 30 kDa) and at 2% of the peak mass for spectra optimized for high mass (30–100 kDa). Qualified mass peaks (S/N > 5) within m/z range of 2.5–100 kDa were auto detected. Peak clustering was completed using a second-pass peak selection (S/N > 2, within 0.3% mass window) and estimated peaks added.

Biomarker purification

For marker purification, pooled fractions containing the biomarker of interest were subjected to further 2D fractionation by combining Free Flow Electrophoresis (FFE) and GE.

FFE separation was conducted in the IEF mode (IEF-FFE) using a BD FFE System (BD Diagnostics, Sparks, MD, USA) as described previously (33;34). Separation of pooled SAX-fractionated serum samples was performed across a pH range of 3–10. The separated sample was collected (3.5 ml/fraction) to a 96-well plate with a separation time of 1.5 h. Of the total fractions (n = 96) collected from FFE separations, 88 were analyzed for total protein separation by GE.

Consecutive FFE fractions were pooled and then dried by vacuum centrifugation. Proteins and/or polypeptides were suspended in 50 µL of 2× gel-loading buffer (100 mM Tris–HCl pH 6.8, 4% (w/v) SDS, 0.2% (w/v) bromophenol blue, 20% (v/v) glycerol, and 200 mM 2-mercaptoethanol) and heated at 95°C for 5 minutes to denature the proteins and/or polypeptides. Protein and/or polypeptides were further separated utilizing PAGE through a (4/10/16%) Tricine/SDS/polyacrylamide gel at 100 V for 4 hours at room temperature.

Following electrophoresis, the gel was silver stained using a Silverquest staining kit (Invitrogen, Paisley, UK; LC6070) and bands near the size of the putative biomarker

were excised from the gel using 1.2 mm Harris Unicore punches (Ted Pella, Redding, CA, USA).

In-gel Trypsin Digestion

Candidate silver stained bands representing the marker of interest, blank gel pieces from a spot-free region, and reference spots (known marker proteins from the gel ladder) were excised and placed in a 96-well plate. In-gel digestion was performed automatically using a ProGest robotic system (DIGILAB, Holliston, MA, USA) following standard digestion protocols (35). The gel plugs were digested for 4 hours at 37°C and the reaction was stopped by adding 7 µL of 3% formic acid to each well. The supernatant was removed. Extracted peptides were reconstituted in 25 µL 0.1% TFA for tandem mass analysis.

Infusion analysis with high resolution FT-Orbitrap MS^2 Analysis

Infusion experiments were performed on an LTQ Orbitrap Elite system (Thermo Fisher Scientific, Bremen, Germany). The isolated lyophilized tryptic peptide fractions were freshly reconstituted in 1 mL solution containing 2% ACN, 0.5% Formic acid and infused at 150 nL/min onto the Nanospray Flex nESI source (Thermo Fisher Scientific, Wien, Austria) and connected to a 1P-4P coated, 8 m tip × 360 µm OD × 75 µm ID PicoTip™ nESI emitter (New Objective, Dingoes, NJ, USA). The LTQ-Orbitrap settings were as follows: spray voltage 1.5 kV; full MS mass range: *m*/*z* 300 to 2 000, operated in positive ion mode with data-dependent acquisition. A single full-scan MS in the Orbitrap (120 000 resolution, 300–2 000 m/z) was followed by six data-dependent MS^2 scans for precursor ions above a threshold ion count of 10 000, using the multipole device (HCD; higher collision energy dissociation) with the resolution set to 7 500 and 45% normal collision energy. The data files (x.raw) were converted into mascot generic files using the MassMatrix File Conversion Tool (Version 2.0; http://www.massmatrix.net) for input into the Mascot searching algorithm (Matrix Science). The data files were searched against SwissProt (v. 2010_06) NCBInr (v. 20080527) using the following search criteria: tryptic peptides with up to one missed cleavage and carbamidomethylation of cysteines and oxidation of methionines, which were set as variable modifications.

LC MS^2 analysis

Peptides were analyzed by LC MS/MS using a Surveyor LC system and LCQ Deca XP Plus (ThermoScientific). Briefly, peptides were resolved by reverse phase chromatography (Biobasic column, ThermoScientific; 180 µM × 15 mm) over a 30 minute ACN gradient at a flow rate of 2 µL/minute. Peptides were ionized by electrospray ionization (ESI) and were subjected to product ion MS^2 analysis. Mass accuracy, resolution and sensitivity of the ESI-MS^2 component were confirmed with the direct infusion of glufibrinopeptide (2.5 pMol/µL). Optimum LC MS/MS system performance for retention time reproducibility, mass accuracy, sensitivity, and protein sequence coverage were verified by the peptide analysis of a BSA tryptic digest (500 pg/µL). BSA quality control checks were performed before and after analysis of experimental samples. The data extraction and processing tasks were the same with those of the infusion FT-Orbitrap experiment.

Biomarker verification by immunoprecipitation (IP) MS

Serum fractions containing ANX A3 were immunodepleted using the µMACS protein A/G microbeads kit (Miltenyi Biotec, Bergisch Gladbach, Germany; 130-042-601) according to the manufacturer instructions. In brief, 2 µL of monoclonal ANX A3 antibody (Santa Cruz Biotechnology, Santa Cruz, CA, USA; sc-134260) was added to 100 µL of protein A/G and then mixed with 200 µL of sample before incubation on ice for 30 minutes. The mix was loaded onto a magnet mounted µMACS column followed by 4 washes with PBS-T. Purified protein was eluted by ACN/TFA/Isopropranol (16.7/0.1/33.3%–50%).

ANX A3 ELISA validation

ANX A3 measurements in serum fractions and cell lysate/media were performed using commercially available ANX A3 sandwich ELISA kits according to the manufacturers' protocol (USCN Life Science, E94786Hu and CUSABIO Biotech Co. CSB-E12157 h). The total protein concentration of each cell line was determined using a BCA protein assay (Pierce, Thermo Scientific, Rockford, IL, USA) and equal protein quantities were used in the analyses. All samples and standards were run in duplicate with absorbance measured on a ThermoMax plate reader (Molecular Devices, Sunnyvale, CA, USA) and data analyzed using GraphPad Prism 5 software. Mann Whitney *U*-Tests were used to analyze all ELISA results and a $P < 0.05$ reflected a significant difference between groups.

Breast tissue Immunohistochemistry

Immunohistochemistry was carried out on 4 µm thick tissue sections cut from formalin fixed paraffin embedded tissue blocks using a Microm HM 325 microtome (Thermo Scientific, Walldorf, Germany) and

mounted onto Superfrost ® Plus coated slides (Thermo Scientific). Sections were incubated with monoclonal; ANX A3 antibody (tgc Biomics, Mainz, Germany; clone tgc7 ProVII5C5). Immunohistochemistry was performed using a Bond-Max fully automated immunostaining machine with Bond reagents (Leica Microsytems, Milton Keynes, UK). Sections were dewaxed, pretreated, stained and counterstained using the standard Bond protocol. Slides were pretreated using the Bond ER2 protocol (20 minutes of EDTA). Primary antibody was then applied at a working dilution of 1:1 000, with an incubation period of 20 minutes. A peroxidase blocking step was included following primary antibody incubation. The Bond autostainer used a biotin-free, polymeric horseradish peroxidase-linked antibody conjugate detection system (Bond™ Polymer Refine Detection System, Leica Microsystems, Milton Keynes, UK) with a DAB-chromogen. The slides were counterstained with haematoxylin and mounted under glass coverslips in Pertex (Histolab® products, Gothenburg, Sweden) and imaged using a Leica CV5330 machine (Leica Biosystems, UK).

The frequency of positive ANX A3 staining was assessed using a four point scoring system: 0 for no positive cells, 1 for 1–25% of positive cells, 2 for 26–50% of positive cells and 3 for 51–100% of positive cells. In addition, an intensity score of positive staining was also used: 0 (negative staining), 1 (weak, light beige staining), 2 (moderate, brown staining), and 3 (strong, brown staining). An ANX A3 expression index was calculated as follows: expression index = Positive staining score X Intensity score. High ANX A3 expression was defined as an expression index score of > 4, and low expression defined as an expression index score of < 4. Two independent pathologists, blinded with respect to the clinic-pathological information, performed the scoring. The X^2 test was used to evaluate differences in ANX A3 expression between two groups of tissues.

Breast cells, lysates and media analysis

The six neoplastic human breast cell lines (HUMA121, MCF7, T47D, MDA MB231, SKBR3 and ZR 75–1) that were used in this study were cultured in appropriate media and incubated in a humidified atmosphere containing 5% CO2 at 37°C. The HUMA121 cells were cultured in RPMI (Gibco BRL, NY, USA) with 10% FBS, the remaining cell lines were cultured in Dulbecco's Modified Eagle Medium (DMEM; Gibco BRL, NY, USA) supplemented with 10% FBS. The media was removed once cells reached 70–80% confluence; cells were rinsed twice with 10 ml of PBS and then 30 ml of chemically defined complete hamster ovary (Gibco BRL, NY, USA) media was added followed by further 24 hours incubation. The conditioned media were collected and dialyzed for 48 hours in ultrapure water (with one water change after 24 hours) using a 2 kDa cut off dialysis cassette (Pierce, Thermo Scientific, Rockford, IL, USA). The cells were trypsinized and counted before being lysed with RIPA buffer. Protein concentrations were determined using a BCA kit (Pierce. Cat. No 23227). Equal total protein concentrations from conditioned media and cell lysates were analyzed by ANX A3 ELISA.

Immunoblotting

Cells were harvested using trypsin digestion and lysed using RIPA buffer with protease inhibitor cocktail (Sigma, P8340). Total protein was quantified using BCA protein assay kit (Pierce. Cat. No 23227) and equal quantities of denatured protein were subjected to electrophoresis on SDS-polyacrylamide gels, blotted onto Immobilon-FC transfer membrane and probed with specific primary antibodies and secondary antibodies. Primary antibodies used: ANX A3 (Santa Cruz Biotechnology, sc101885) and GAPDH (Abcam, ab8245). Secondary antibodies used: Goat anti-mouse IRDye 800CW (IRDye Antibodies, 926–32211) and Goat anti-rabbit IgG IRDye 680LT (IRDye Antibodies, 926–68021). The signal was visualized using a LICOR Odyssey® image system.

Small interfering RNA (siRNA)

ANX A3-siRNA (Santa Cruz Biotechnology, sc-89288) was used to silence *ANX A3* expression and ON-TARGET plus non-targeting siRNA #1 (Dharmacon, D-0018100105) was used as a negative control. MCF7 cells were transfected with siRNA using Oligofectamine™ (Invitrogen) as per manufacturer's instructions.

Transwell migration assay

BD bioscience transwell assays were used as per the manufacturer's instructions. Briefly, wells and inserts were equilibrated by adding DMEM medium supplemented with 10% FCS at least 30 minutes prior to cell seeding. MCF7 cells were seeded into inserts in DMEM medium supplemented with 10% FCS at a density of 5 × 10^5 cells per transwell insert so that a fully confluent cell layer was formed. After cells had adhered (approximately 6 hours post seeding) medium in inserts was removed and replaced with DMEM with no FCS. At indicated time points cells within the transwell and those that had migrated to the underside of the cell permeable membrane were washed with PBS

Supplementary Table S1: The clinicopathological characteristics of the Wessex breast cancer cohort

Clinical and Pathologic Features of the Wessex cohort samples	
**Characteristics**	**Percentage**
**Diagnostic category**	
Control	630 (60.5%)
BBD	192 (18.4%)
IDC	219 (21%)
**Age median [range] (SD)**	63.6 [50.8–75.1] (7.33)
**Menopausal status**	
Pre-menopause	186 (18%)
Peri-menopause	402 (38.6 %)
Post-menopause	417 (40.1%)
Unknown	36 (3.5%)
**Benign disease sub groups**	
Microcalcifications	21 (2%)
Duct adenosis	22 (2.1%)
Fibroadenoma	50 (4.8%)
Fibrocystic changes	41 (3.9%)
Typical hyperplasia	9 (0.9%)
Sclerosing adenosis	6 (0.6%)
Cyst	21 (1.9%)
ADH	0 (0%)
Others	24 (2.3%)
**DCIS element**	51 (4.9%)
ER negative	51 (4.9%)
ER Positive	108 (10.4%)
**IDC**	
IDC	132 (12.7%)
ILC	0 (0%)
Mixed	87 (8.4%)
**Grade***	
Low	306 (29.4%)
Intermediate	105 (10.1%)
High	99 (9.5%)
**ER**	
Positive	177 (17.0%)
Negative	12 (1.2%)

Clinical and Pathologic Features of the Wessex cohort samples	
**Characteristics**	**Percentage**
Unknown	30 (2.9%)
**PR**	
Positive	72 (6.9%)
Negative	12 (1.2%)
**HER2**	
Positive	92 (9.2%)
Negative	69 (6.6%)
**Lymph node involvement**	
Negative	78 (7.5%)
Positive	48 (4.6%)

and fixed with ice cold acetone:methanol 1:1 fixative. The cells within the transwell were stained with eosine solution and removed using a cotton bud and thorough removal was confirmed under a microscope. Cells on the underside of the of the cell permeable membrane were washed, stained with DAPI and representative fields of view were imaged using an Olympus CKX41 fluorescence microscope and cells counted using ImageJ software.

Proliferation assay

Cells were seeded at density of 5000 per well of 24 well plates at indicated time points media was removed, cells were washed once with PBS and then fixed with ice cold acetone:methanol 1:1 fixative. Cells were then stained with DAPI, representative fields of view were imaged using an Olympus CKX41 fluorescence microscope and cells counted using ImageJ software.

FILE S2

Mascot Search Results

Protein View

Match to: **ANXA3_HUMAN** Score: **259**
Annexin A3 OS=Homo sapiens GN=ANXA3 PE=1 SV=3
Found in search of C:\Documents and Settings\Administrator\Desktop\Helens\Guy Whiteley\Bash5.mgf

Nominal mass (M_r): **36353**; Calculated pI value: **5.63**
NCBI BLAST search of ANXA3_HUMAN against nr
Unformatted sequence string for pasting into other applications

Taxonomy: Homo sapiens

Variable modifications: Carbamidomethyl (C),Oxidation (M)
Cleavage by Trypsin: cuts C-term side of KR unless next residue is P
Sequence Coverage: **40%**

Matched peptides shown in **Bold Red**

1 MASIWVGHRG TVR**DYPDFSP SVDAEAIQK**A IRGIGTDEK**M LISILTER**SN
51 AQRQLIVKEY QAAYGKELKD DLKGDLSGHF EHLMVALVTP PAVFDAKQLK
101 KSMK**GAGTNE DALIEILTTR** TSRQMK**DISQ AYYTVYK**KSL **GDDISSETSG**
151 **DFRKALLTLA DGR**RDESLKV DEHLAK**QDAQ ILYK**AGENRW GTDEDKFTEI
201 LCLRSFPQLK **LTFDEYR**NIS QK**DIVDSIK**G ELSGHFEDLL LAIVNCVR**NT**
251 **PAFLAER**LHR ALK**GIGTDEF TLNR**IMVSRS EIDLLDIRTE FKKHYGYSLY
301 SAIK**SDTSGD YEITLLK**ICG GDD

Supplementary Figure S2: Tandem mass sequence coverage of the identified serum biomarker ANX A3. Tryptic peptides determined by product ion LC-ESI MS/MS analysis.

FILE S3

Sequence: GAGTNEDALIEILTTR, G1-iTRAQ8plex (304.20536 Da)
Charge: +2, Monoisotopic m/z: 989.54498 Da (+3.4 mmu/+3.43 ppm), MH+: 1978.08269 Da, RT: 37.20 min,
Identified with: Mascot (v1.15); IonScore:79, Exp Value:4.0E-007, Ions matched by search engine: 13/164
Fragment match tolerance used for search: 0.8 Da
Fragments used for search: b; b-H_2O; b-NH_3; y; y-H_2O; y-NH_3
Protein references (1):

- Annexin A3 OS=Homo sapiens GN=ANXA3 PE=1 SV=3 - [ANXA3_HUMAN]

#1	b^+	b^{2+}	Seq.	y^+	y^{2+}	#2
1	362.23411	181.62069	G-iTRAQ8plex			16
2	433.27123	217.13925	A	1616.84907	808.92817	15
3	490.29270	245.64999	G	1545.81195	773.40961	14
4	591.34038	296.17383	T	1488.79048	744.89888	13
5	705.38331	353.19529	N	1387.74280	694.37504	12
6	834.42591	417.71659	E	1273.69987	637.35357	11
7	949.45286	475.23007	D	1144.65727	572.83227	10
8	1020.48998	510.74863	A	1029.63032	515.31880	9
9	1133.57405	567.29066	L	958.59320	479.80024	8
10	1246.65812	623.83270	I	845.50913	423.25820	7
11	1375.70072	688.35400	E	732.42506	366.71617	6
12	1488.78479	744.89603	I	603.38246	302.19487	5
13	1601.86886	801.43807	L	490.29839	245.65283	4
14	1702.91654	851.96191	T	377.21432	189.11080	3
15	1803.96422	902.48575	T	276.16664	138.58696	2
16			R	175.11896	88.06312	1

#1	b^+	b^{2+}	Seq.	y^+	y^{2+}	#2
1	+0.85	-	G-iTRAQ8plex			16
2	+0.45	-	A	–9.85	-	15
3	-8.33	-	G	–9.04	-	14
4	+1.52	-	T	+8.99	-	13
5	+2.69	-	N	–2.90	-	12
6	+4.61	-	E	–12.71	-	11
7	–2.92	+1.47	D	–4.55	-	10
8	–4.02	–0.48	A	–4.55	-	9
9	–0.90	+10.14	L	–14.83	-	8
10	–4.97	-	I	–4.14	-	7
11	–7.08	-	E	–6.24	-	6
12	+5.16	-	I	–3.01	-	5
13	-	-	L	+3.29	-	4
14	-	-	T	–2.91	-	3
15	-	-	T	–2.60	-	2
16			R	–3.48	-	1

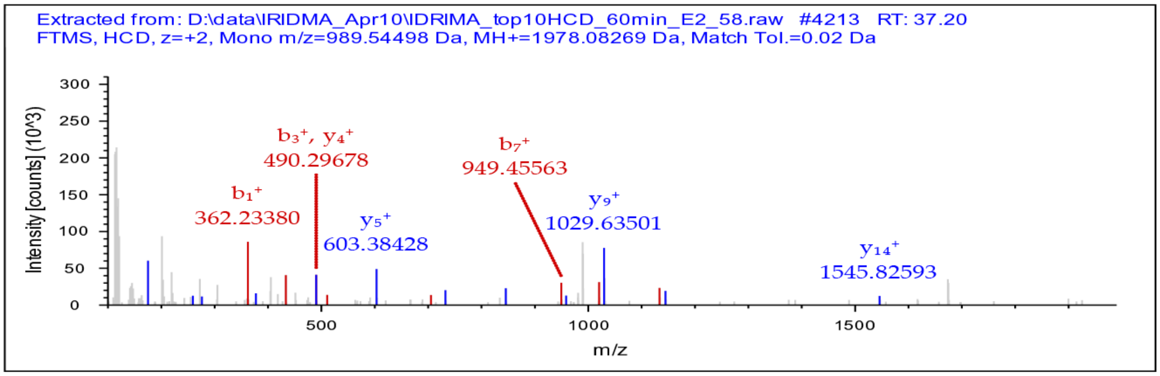

Sequence: SLGDDISSETSGDFR, S1-iTRAQ8plex (304.20536 Da)
Charge: +2, Monoisotopic m/z: 945.45715 Da (+1.98 mmu/+2.09 ppm), MH+: 1889.90703 Da, RT: 29.29 min,
Identified with: Mascot (v1.15); IonScore:63, Exp Value:1.7E-005, Ions matched by search engine: 10/136
Fragment match tolerance used for search: 0.8 Da
Fragments used for search: b; b-H_2O; y; y-H_2O; y-NH_3

Protein references (1):
- Annexin A3 OS=Homo sapiens GN=ANXA3 PE=1 SV=3 - [ANXA3_HUMAN]

#1	b^+	b^{2+}	Seq.	y^+	y^{2+}	#2
1	392.24467	196.62597	S-iTRAQ8plex			15
2	505.32874	253.16801	L	1498.66568	749.83648	14
3	562.35021	281.67874	G	1385.58161	693.29444	13
4	677.37716	339.19222	D	1328.56014	664.78371	12
5	792.40411	396.70569	D	1213.53319	607.27023	11
6	905.48818	453.24773	I	1098.50624	549.75676	10
7	992.52021	496.76374	S	985.42217	493.21472	9
8	1079.55224	540.27976	S	898.39014	449.69871	8
9	1208.59484	604.80106	E	811.35811	406.18269	7
10	1309.64252	655.32490	T	682.31551	341.66139	6
11	1396.67455	698.84091	S	581.26783	291.13755	5
12	1453.69602	727.35165	G	494.23580	247.62154	4
13	1568.72297	784.86512	D	437.21433	219.11080	3
14	1715.79139	858.39933	F	322.18738	161.59733	2
15			R	175.11896	88.06312	1

#1	b⁺	b²⁺	Seq.	y⁺	y²⁺	#2
1	+2.52	-	S-iTRAQ8plex			15
2	–3.51	-	L	+5.32	-	14
3	+4.00	-	G	–2.24	-	13
4	+1.56	-	D	-	-	12
5	–1.24	-	D	–0.51	-	11
6	–4.63	-	I	–5.88	-	10
7	-	-	S	+0.73	-	9
8	-	-	S	+2.86	-	8
9	-	-	E	+1.83	-	7
10	-	-	T	–4.18	-	6
11	-	-	S	+2.43	-	5
12	-	-	G	-	-	4
13	-	-	D	-	-	3
14	-	-	F	–3.60	-	2
15			R	–3.19	-	1

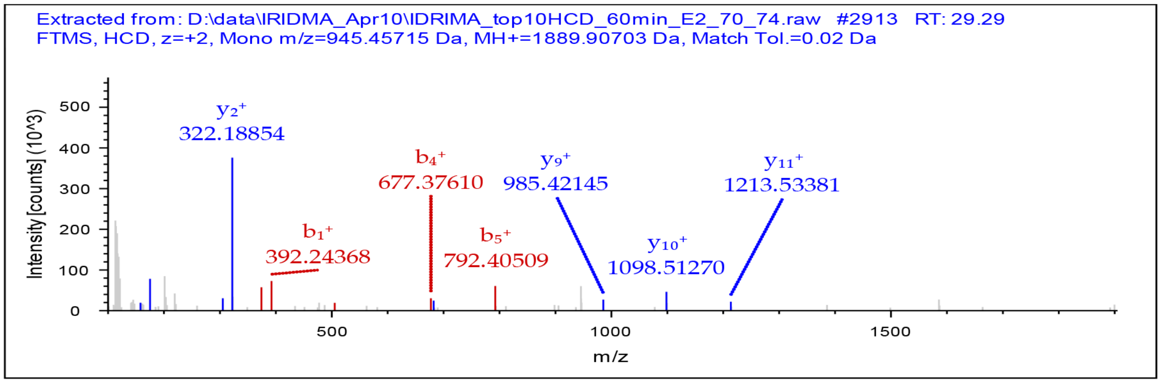

Sequence: MLISILTER, M1-iTRAQ8plex (304.20536 Da)
Charge: +2, Monoisotopic m/z: 690.41199 Da (-3.36 mmu/-4.87 ppm), MH+: 1379.81670 Da, RT: 36.68 min,
Identified with: Mascot (v1.15); IonScore:29, Exp Value:1.2E-002, Ions matched by search engine: 8/72
Fragment match tolerance used for search: 0.8 Da
Fragments used for search: b; b-H_2O; y; y-H_2O; y-NH_3

Protein references (1):
- Annexin A3 OS=Homo sapiens GN=ANXA3 PE=1 SV=3 - [ANXA3_HUMAN]

#1	b^+	b^{2+}	Seq.	y^+	y^{2+}	#2
1	436.25314	218.63021	M-iTRAQ8plex			9
2	549.33721	275.17224	L	944.57755	472.79241	8
3	662.42128	331.71428	I	831.49348	416.25038	7
4	749.45331	375.23029	S	718.40941	359.70834	6
5	862.53738	431.77233	I	631.37738	316.19233	5
6	975.62145	488.31436	L	518.29331	259.65029	4
7	1076.66913	538.83820	T	405.20924	203.10826	3
8	1205.71173	603.35950	E	304.16156	152.58442	2
9			R	175.11896	88.06312	1

#1	b^+	b^{2+}	Seq.	y^+	y^{2+}	#2
1	+9.00	-	M-iTRAQ8plex			9
2	+6.09	-	L	+4.24	-	8
3	+8.22	-	I	+3.25	-	7
4	+10.18	-	S	+3.63	-	6
5	+9.65	-	I	+6.57	-	5
6	-	-	L	+3.96	-	4
7	-	-	T	+5.68	-	3
8	-	-	E	–0.78	-	2
9			R	+2.98	-	1

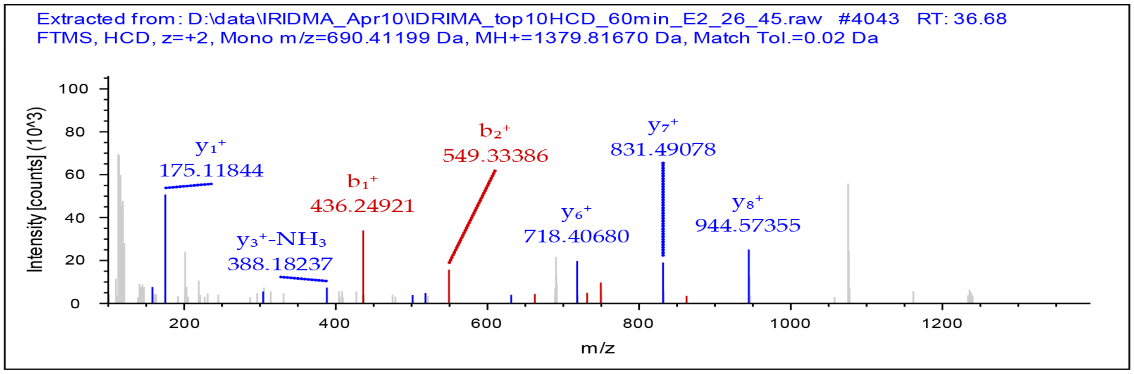

Supplementary Figure S3: Annotated Annexin A3 tryptic peptide product ion spectra obtained with the infusion FT-Orbitrap MS²analysis. Tabulation of the β- and γ- ions observed for all three unique tryptic peptide observed are listed alongside the high resolution product ion spectra.
